# Real-World Effectiveness of Ravulizumab Among C5 Inhibitor-Naive Patients With Atypical Hemolytic Uremic Syndrome: A Physician Panel-Based Chart Review (aHUS IMPACT Study)

**DOI:** 10.1016/j.xkme.2025.101198

**Published:** 2025-12-09

**Authors:** Ramy Magdy Hanna, Shruti Chaturvedi, Moh-Lim Ong, Arpita Nag, Rui Song, Lynn Huynh, Jordan A. Burdeau, Mei Sheng Duh, Yan Wang

**Affiliations:** 1 University of California, Irvine, CA; 2 Johns Hopkins University, Baltimore, MD; 3 Alexion, AstraZeneca Rare Disease, Boston, MA; 4 Analysis Group Inc, Boston, MA

**Keywords:** Atypical hemolytic uremic syndrome, ravulizumab, complement C5 inhibitor, thrombotic microangiopathy, adult, real world

## Abstract

**Rationale & Objective:**

Atypical hemolytic uremic syndrome (aHUS) is a rare form of thrombotic microangiopathy (TMA) caused by complement dysregulation. Ravulizumab, a complement C5 inhibitor (C5i), is approved for aHUS; however, published evidence in a real-world setting is limited.

**Study Design:**

Retrospective, longitudinal, physician panel-based chart review.

**Setting & Population:**

C5i-naive adults with aHUS in the United States treated with ravulizumab. Physicians randomly selected 1-5 patients who had ≥6 months of follow-up after ravulizumab initiation; patients who died within 6 months of initiation were eligible.

**Exposure(s) or Predictor(s):**

Ravulizumab.

**Outcomes:**

The clinical outcomes evaluated included hematologic and renal outcomes, complete TMA response (a composite hematolgic/renal endpoint), and dialysis use.

**Analytical Approach:**

Descriptive statistics, Kaplan-Meier estimators, and generalized linear models.

**Results:**

Overall, 79 C5i-naive adults with aHUS (enrolled by 31 physicians) initiated ravulizumab and were included in the study. Statistically significant improvements from baseline occurred as early as day 4 (lactate dehydrogenase and percent change in serum creatinine; both *P* < 0.001) and day 8 (platelet count; *P* < 0.001). The proportions of patients with normalization of platelet counts and lactate dehydrogenase levels, and ≥25% improvement in serum creatinine levels, were 14 out of 67 (21%), 12 out of 58 (21%), and 10 out of 65 (15%) at day 4, and 40 out of 48 (83%), 35 out of 38 (92%), and 42 out of 48 (88%) at 12 months after ravulizumab initiation, respectively. Complete TMA response rates were 60% and 68% within 6 and 12 months after ravulizumab initiation, respectively, and the median (interquartile range) time to complete TMA response was 3.1 (1.0-14.0) months. Of the 20 patients who received any dialysis at baseline, 14 (70.0%) did not have dialysis during follow-up.

**Limitations:**

The study design relies on available medical record data and has potential responder bias.

**Conclusions:**

This study supports the immediate and sustained benefits of initiating ravulizumab in patients with aHUS as seen by the early response and continued improvement in clinical outcomes.

## Plain-Language Summary

This study evaluates the use of a treatment called ravulizumab for atypical hemolytic uremic syndrome (aHUS), a rare disease that causes clots in small blood vessels and can lead to kidney failure. Researchers reviewed the medical records of 79 adults with aHUS in the United States who were treated with ravulizumab and had never used similar treatments before. Significant improvements in laboratory measures including lactate dehydrogenase and serum creatinine levels occurred by day 4, and in platelet count by day 8. At month 12, most patients had normalized platelet counts and lactate dehydrogenase levels, and improved serum creatinine levels. Many patients were able to stop receiving dialysis during the follow-up period. The results suggest that ravulizumab provides immediate and lasting benefits for patients with aHUS.

Atypical hemolytic uremic syndrome (aHUS) is a rare form of thrombotic microangiopathy (TMA) caused by complement dysregulation.^1^^,^^2^ It is a progressive disease that can involve multiple extrarenal complications and, without effective treatment, it can result in severe organ damage and death.^1^

Management of aHUS has substantially improved since the introduction of therapies that inhibit complement C5 activation and thus block the formation of complement C5b-9, the membrane attack complex that drives the cellular damage underlying the disease.^3^ Eculizumab, a humanized monoclonal antibody that blocks terminal complement activation at C5, was the first approved treatment for patients with aHUS and changed the natural history of the disease.^2^^,^^4^, ^5^, ^6^, ^7^, ^8^ Ravulizumab is a next-generation terminal complement inhibitor that has an extended half-life and weight-based dosing, allowing for more precise inhibition across body weights with fewer infusions compared with eculizumab.^9^ Ravulizumab is approved for the treatment of aHUS^10^^,^^11^ on the basis of evidence from registrational clinical studies, in which immediate, complete, and sustained C5 inhibition was demonstrated.^12^, ^13^, ^14^

Although there is some case literature reporting on ravulizumab treatment in patients with aHUS naive to C5 inhibitors,^15^, ^16^, ^17^, ^18^, ^19^ there are no robust data on the real-world effectiveness of ravulizumab in patients who have not previously received complement inhibitor treatment. Therefore, the aim of this study was to assess the real-world effectiveness of ravulizumab on clinical outcomes—including complete TMA response, hematologic and renal outcomes, and dialysis use—in C5 inhibitor-naive patients with aHUS.

## Methods

### Study Design

**Figure 1:**
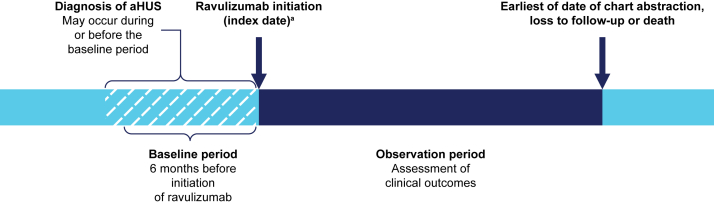
Study design. ^a^Initiation of the first complement inhibitor treatment (ravulizumab) between November 1, 2019, and September 30, 2022. Abbreviation: aHUS, atypical hemolytic uremic syndrome.

This was a retrospective, longitudinal, US physician panel-based chart review study. The study design is summarized in Fig 1. The index date was defined as the date of initiation of the first complement inhibitor treatment with ravulizumab, during the period from November 1, 2019, to September 30, 2022. The baseline period was defined as the 6-month period before the index date. The diagnosis of aHUS could occur during or before the baseline period. Patient demographics and clinical characteristics were described on the index date, or during the baseline period as close as possible to the index date. Patients’ medical histories after aHUS diagnosis, which was not restricted to the baseline period (eg, kidney transplant), were also collected. The observation period was defined as the time from the index date to the earliest of date of chart abstraction, loss to follow-up, or death.

The primary objectives were to evaluate hematologic (blood platelet counts, serum lactate dehydrogenase [LDH] levels, and hemoglobin levels) and renal parameters (serum creatinine [SCr] levels or estimated glomerular filtration rate [eGFR]) at selected timepoints (4, 8, and 15 days and 1, 3, 6, and 12 months) after initiation of ravulizumab, compared with baseline. Secondary objectives were to describe patient demographics, clinical characteristics, treatment patterns, patient dialysis use, and the proportion of patients who achieved complete TMA response as well as the time to complete TMA response.

Institutional Review Board review and ethics approval for this study were waived under the exempt research category because data were anonymized and patient informed consent was not required.

### Study Population

Eligible physicians were nephrologists, hematologists, or hematologist-oncologists with access to complete medical records for at least 1 patient with a diagnosis of aHUS who received ravulizumab. Access to key laboratory readings (including platelet counts and LDH, SCr, eGFR, and hemoglobin levels) was also required. US physicians meeting the study inclusion criteria were invited to participate in the study and to randomly select up to 5 eligible patients whose charts would be abstracted. In addition to a confirmed diagnosis of aHUS, eligible patients were required to have initiated ravulizumab as a first complement inhibitor treatment between November 1, 2019, and September 30, 2022, to be aged ≥18 years at the time of ravulizumab initiation, and to have at least 6 months of follow-up data after the index date, unless the patient died during these 6 months. Patients were excluded if, anywhere in their available medical history, they had documented hemolytic uremic syndrome related to known defects of cobalamin C metabolism, a previous diagnosis of Shiga toxin-producing *Escherichia coli*-associated hemolytic uremic syndrome, a previous diagnosis of thrombotic thrombocytopenic purpura, or receipt of ravulizumab and/or eculizumab in a clinical trial.

### Data Collection

A third-party physician panel vendor (Medefield America Limited LLC) contacted physicians in their network via email to invite them to participate in the study; all physicians had previously consented to being contacted for research participation invitations. An online questionnaire was used to screen for eligibility. Participating physicians were asked to complete a physician characteristic survey and to collect information from randomly selected patients. A computer-generated list of letters was created; eligible physicians were then instructed to select a patient whose last name started with the corresponding letter and extract chart information for that patient. Patient data were collected and reported via an electronic case report form including both structured and unstructured fields, using an online portal. The study was double blinded between the study sponsor and enrolled physicians, and patient data were anonymized.

The electronic case report form was pilot tested (1 nephrologist and 1 hematologist-oncologist in the United States) to ensure that all questions were clear and correctly interpreted. During the pilot test, physicians could comment on questions they did not understand, request clarifications, and provide suggestions to improve questions. After this, a soft launch with 5% of eligible physicians was conducted to evaluate the quality of the data. The full launch commenced after all quality issues were resolved.

For analysis of the primary objective, platelet counts, LDH levels, and SCr or eGFR levels were collected at baseline and within a window of ±1 day of days 4, 8, and 15 after index, and within a window of ±2 weeks of months 1, 3, 6, and 12 after index. For analysis of the secondary objective, complete TMA response was recorded during the observation period on the basis of physician reports or laboratory measures. Complete TMA response was defined as platelet count normalization (≥150 × 10^9^/L), LDH normalization (≤246 U/L), and improvement in kidney function (≥25% reduction in SCr from baseline). Dialysis was classified as acute (for acute kidney injury) or chronic (on a regular basis as renal replacement therapy for end-stage kidney disease) and was recorded for the time periods from 12 months before the index date to 2 weeks after the index date and from 2 weeks to 12 months after the index date. Additional recorded variables were patient demographics, disease and clinical characteristics, treatment patterns, reasons for treatment discontinuation, and physician characteristics.

### Statistical Analysis

Statistical analyses were performed using Statistical Analysis Software Enterprise Guide Version 7.15 or its latest version (Statistical Analysis Software Institute Inc).

For primary objectives, generalized linear models with a normal distribution and an identity link were used, that is, outcome variables were modeled directly on their original scale without transformation. Generalized estimating equations were applied to account for within-patient correlation owing to repeated measurements when comparing postindex laboratory measures with baseline. Log transformation was conducted for SCr values to improve normality of the distribution. The mean difference of platelet count and LDH level, and the percentage change for SCr in post index timepoints compared with baseline, were reported with corresponding 95% confidence intervals (CIs) and *P* values.

For secondary objectives, categorical variables were summarized with frequencies and proportions. Continuous variables were summarized with means and standard deviations or medians and interquartile ranges (IQRs). The proportion of patients with complete TMA response was recorded within 6 and 12 months after the index date. Time to complete TMA response was evaluated using Kaplan-Meier estimators.

A sensitivity analysis of patients without missing data for platelet counts, LDH levels, and SCr levels reported at day 4, day 8, month 6, and month 12 was conducted.

## Results

### Physician and Patient Characteristics

In total, 31 physicians were enrolled and provided patient charts; key physician characteristics are summarized in Table S1. The most common medical specialty was hematologist-oncologist (61.3%), and more than half (58.1%) of the physicians had been practicing for 11-20 years. Overall, 25 out of 31 (80.6%) physicians reported having diagnosed and/or managed at least 5 adult patients with aHUS each year, and among the physicians who also managed pediatric patients, 11 out of 19 (57.9%) physicians diagnosed and/or managed at least 5 pediatric patients each year.

Overall, the physicians provided charts for 79 adult C5 inhibitor-naive patients with aHUS who had initiated ravulizumab with a median (IQR) follow-up duration of 24.8 (19.9-38.4) months (Table 1). The median (IQR) age at ravulizumab initiation was 44.4 (26.8-54.1) years, and 59.5% were men. Overall, 19.0% of patients had a family history of aHUS. In total, 3 (3.8%) patients had received a kidney transplant before initiating ravulizumab (median [IQR] time from kidney transplant to the index date, 3.4 [2.1-4.6] months). These 3 patients had existing comorbid conditions including hypertension (n=2), chronic kidney disease (n=2), diabetes (n=1), and congestive heart failure (n=1). Genetic variants were detected in approximately three-quarters of patients in whom genetic tests were performed (35/45 [77.8%]). Among patients who underwent genetic testing, the most commonly identified variant was complement factor H (14/45; 31.1% patients). In total, 47 (59.5%) patients had a known aHUS trigger, 18 (22.8%) patients had no trigger, and 14 (17.7%) patients had an unknown trigger status (Table S2). Among patients with an autoimmune trigger event, genetic variants were detected in most patients for whom genetic tests were performed (n=15/18 [83.3%]; Table S3).

**Table 1:** Patient Demographic and Clinical Characteristics

Characteristic	All Patients (N=79)
Age, y, median (IQR)	
Age at first confirmed aHUS diagnosis	44.3 (26.7-53.8)
Age at index date^a^	44.4 (26.8-54.1)
Sex, n (%)	
Male	47 (59.5)
Female	32 (40.5)
Race, n (%)	
White	53 (67.1)
Black or African American	18 (22.8)
Asian	5 (6.3)
Native Hawaiian or other Pacific Islander	1 (1.3)
Unknown	2 (2.5)
Family history of aHUS, n (%)	
Yes	15 (19.0)
No	52 (65.8)
Unknown	12 (15.2)
aHUS trigger events	
Yes	47 (59.5)
No	18 (22.8)
Unknown	14 (17.7)
Genetic mutations, n (%)	
Genetic test(s) performed	45 (57.0)
Any genetic variants detected^b^	35 (77.8)
*CFHR1*	13 (37.1)
*CFH*	14 (40.0)
*CFHR5*	10 (28.6)
*CFHR3*	9 (25.7)
*CD46*	7 (20.0)
*C3*	6 (17.1)
*THBD*	6 (17.1)
*CFB*	5 (14.3)
*CFI*	3 (8.6)
Treatment history	
Median (IQR) time from diagnosis to first complement inhibitor treatment, mo	0.3 (0.1-1.0)
Kidney transplant before to first complement inhibitor treatment, n (%)	3 (3.8)
Received dialysis during baseline period, n (%)^c^	20 (25.3)
Acute^d^	11 (13.9)
Chronic^e^	8 (10.1)
Unknown	1 (1.3)
Other treatments for aHUS before/after index date, n (%)	
Plasma exchange	28 (35.4)/7 (8.9)
Angiotensin-converting enzyme inhibitors	13 (6.5)/12 (15.2)
Immunosuppressants	13 (6.5)/4 (5.1)
Plasma infusion	6 (7.6)/2 (2.5)
None	38 (48.1)/51 (64.6)
Unknown	2 (2.5)/5 (6.3)
Comorbid conditions, n (%)	
Hypertension	37 (46.8)
Chronic kidney disease	12 (15.2)
Diabetes	12 (15.2)
Congestive heart failure	3 (3.8)
Median (IQR) follow-up duration, mo	24.8 (19.9-38.4)

Abbreviations: aHUS, atypical hemolytic uremic syndrome; *C3,* complement component 3; *CD46*, membrane cofactor protein; *CFB*, complement factor B; *CFH*, complement factor H; *CFHR1*, complement factor H-related protein 1; *CFHR3*, complement factor H-related protein 3; *CFHR5*, complement factor H-related protein 5; *CFI,* complement factor I; IQR, interquartile range; *THBD,* thrombomodulin.

a Age on the index date was determined based on year of birth.

b Complement genetic variant testing (whole-exome sequencing) information was collected following aHUS diagnosis. Only the reporting of the following genetic variants was requested in the electronic case report form: *CD46*, *C3, THBD*, *CFHR1*, *CFHR3*, *CFHR5*, *CFH*, *CFI*, and *CFB*.

c Baseline dialysis was measured during the time period from 12 mo before the index date to 2 wk after the index date.

d Acute dialysis was defined as dialysis from acute kidney injury.

e Chronic dialysis was defined as dialysis on a regular basis as renal replacement therapy for end-stage kidney disease.

### Treatment Pattern

Patients initiated ravulizumab at a median (IQR) follow-up duration of 0.3 (0.1-1.0) months after their aHUS diagnosis. Aside from complement inhibitors, the most commonly used treatment for aHUS before the index date was plasma exchange (35.4% of patients); after the index date, angiotensin-converting enzyme inhibitors were the most commonly used treatment (15.2%). After the index date, there was a reduction in the use of plasma exchange among patients to 8.9%. A quarter (25.3%) of all patients received dialysis during the baseline period.

Ravulizumab discontinuation data were available for 71 out of 79 patients. By 6 months after the index date, 5 patients (6.3% of all patients) discontinued ravulizumab, which increased to 20 (25.3%) patients by 12 months after the index date; no further patients discontinued by 18 months after the index date. Among the 20 patients who discontinued ravulizumab over the course of the study, the median (IQR) treatment duration was 8.6 (6.0-9.5) months. The most common reasons for discontinuation were “reached optimal therapy length” (10/20; 50%), “stabilization or normalization of renal function” (9/20; 45%), and “physician decision” (7/20; 35%); because the entry form allowed multiple responses, categories were not mutually exclusive (Table S4).

### Hematologic and Renal Outcomes

**Figure 2:**
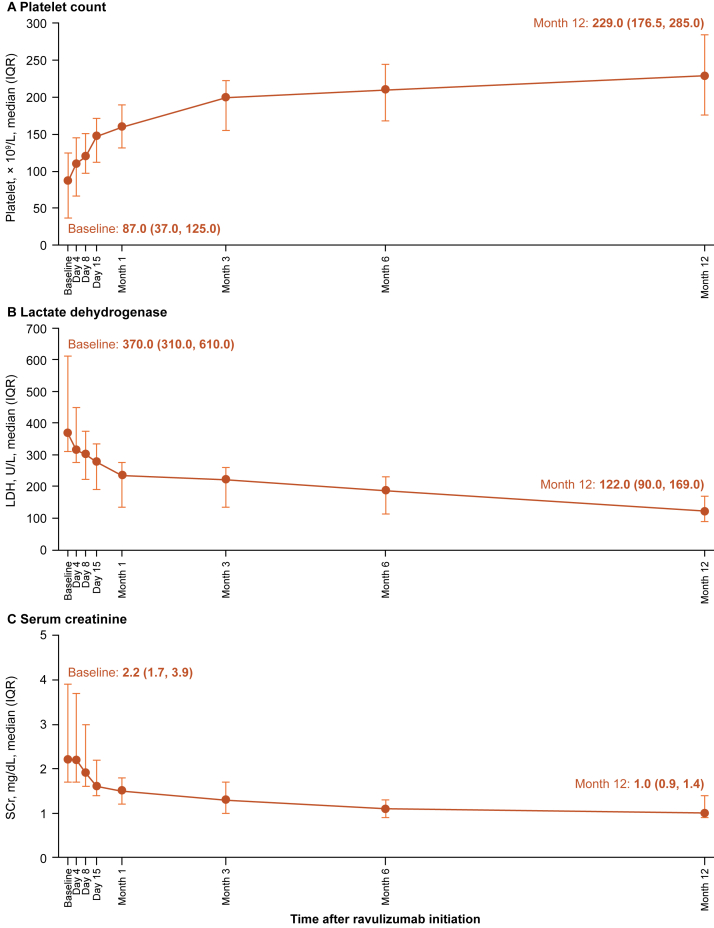
(A) Platelet counts, (B) LDH levels, P (C) SCr levels from baseline to month 12 after ravulizumab initiation. Abbreviations: IQR, interquartile range; LDH, lactate dehydrogenase; SCr, serum creatinine.

Over the 12-month period from baseline, platelet counts increased and levels of LDH and SCr decreased (Fig 2A-C; Table S5). Statistically significant changes from baseline occurred from day 4 in LDH levels (mean difference [95% confidence interval]: -80.5 U/L [-109.3, -51.7]; *P* < 0.001) and SCr levels (percent change [95% confidence interval]: -8.5% [-12.7%, -4.2%]; *P* < 0.001) and from day 8 in platelet counts (mean difference [95% confidence interval]: 27.7 × 10^9^/L [13.2-42.3]; *P* < 0.001) (Table 2). The proportions of patients achieving predefined thresholds for normalization (platelet count and LDH) or improvement (SCr, eGFR, and hemoglobin) are shown in Table 3. At day 4 after the index date, 20.9% and 20.7% of patients achieved normalization of platelet counts (to ≥150 × 10^9^/L) and LDH levels (to ≤246 U/L), respectively. At day 8 after the index date, 32.2% and 33.3% of patients achieved normalization of platelet counts and LDH levels, respectively, and improvements from baseline in SCr (≥25% reduction from baseline), eGFR (≥15 mL/min/1.73 m^2^ increase from baseline), and hemoglobin (≥20 g/L increase from baseline) levels were achieved in 32.8%, 37.8%, and 22.4% of patients, respectively. At 12 months after the index date, 83.3% and 92.1% of patients achieved normalization of platelet counts and LDH levels, respectively, and improvements from baseline in SCr, eGFR, and hemoglobin levels were achieved in 87.5%, 78.6%, and 74.5% of patients, respectively.

**Table 2:** Laboratory Values After Ravulizumab Initiation, Compared With Baseline

Laboratory Parameter (N=79)	Time After Index Date													
	4 d		8 d		15 d		1 mo		3 mo		6 mo		12 mo	
	Estimate (95% CI)	*P* Value^a^	Estimate (95% CI)	*P* Value^a^	Estimate (95% CI)	*P* Value^a^	Estimate (95% CI)	*P* Value^a^	Estimate (95% CI)	*P* Value^a^	Estimate (95% CI)	*P* Value^a^	Estimate (95% CI)	*P* Value^a^
Platelet count, mean difference, × 10^9^/L	12.85 (-2.82, 28.52)	0.11	27.74 (13.22, 42.25)	<0.001	48.46 (31.47, 65.44)	<0.001	62.97 (45.88, 80.06)	<0.001	96.80 (74.05, 119.56)	<0.001	121.08 (94.98, 147.18)	<0.001	148.55 (116.80, 180.30)	<0.001
LDH level, mean difference, U/L	-80.50 (-109.33, -51.66)	<0.001	-145.59 (-191.28, -99.89)	<0.001	-179.31 (-218.92, -139.71)	<0.001	-221.90 (-266.49, -177.31)	<0.001	-253.81 (-302.51, -205.11)	<0.001	-274.16 (-325.07, -223.24)	<0.001	-295.14 (-347.51, -242.78)	<0.001
SCr level, percentage change	-8.54% (-12.66%,-4.23%)	<0.001	-20.59% (-26.38%, -14.34%)	<0.001	-29.94% (-36.50%, -22.70%)	<0.001	-37.99% (-44.06%, -31.26%)	<0.001	-46.89% (-52.60%, -40.50%)	<0.001	-52.50% (-58.03%, -46.25%)	<0.001	-57.03% (-61.67%, 51.84%)	<0.001

Abbreviations: CI, confidence interval; GEE, generalized estimating equation; LDH, lactate dehydrogenase; SCr, serum creatinine.

a Laboratory values before and after the index date were compared via GEE generalized linear models with normal distribution and an identity link.

**Table 3:** Patients With Normalization or Improvement in Laboratory Values Over Time

Laboratory Parameter Threshold Reached, n/N (%) (N=79)	Baseline^a^	Time After Index Date						
		4 d	8 d	15 d	1 mo	3 mo	6 mo	12 mo
Platelet count ≥ 150 × 10^9^/L	9/79 (11.4)	14/67 (20.9)	19/59 (32.2)	33/69 (47.8)	50/74 (67.6)	50/65 (76.9)	54/68 (79.4)	40/48 (83.3)
LDH ≤ 246 U/L	10/77 (13.0)	12/58 (20.7)	17/51 (33.3)	24/61 (39.3)	36/61 (59.0)	38/55 (69.1)	50/58 (86.2)	35/38 (92.1)
SCr reduction ≥ 25% from baseline	-	10/65 (15.4)	19/58 (32.8)	31/68 (45.6)	46/70 (65.7)	53/64 (82.8)	55/67 (82.1)	42/48 (87.5)
eGFR increase ≥ 15 mL/min/1.73 m^2^ from baseline	-	3/48 (6.3)	17/45 (37.8)	26/52 (50.0)	37/53 (69.8)	40/50 (80.0)	43/49 (87.8)	22/28 (78.6)
Hemoglobin increase ≥ 20 g/L from baseline	-	5/66 (7.6)	13/58 (22.4)	23/67 (34.3)	29/70 (41.4)	32/64 (50.0)	47/68 (69.1)	35/47 (74.5)

Abbreviations: eGFR, estimated glomerular filtration rate; LDH, lactate dehydrogenase; SCr, serum creatinine.

a Baseline laboratory values were collected on the index date or at a point closest to the index date within the previous 6 mo.

In a sensitivity analysis of patients without missing data for platelet counts and LDH and SCr levels reported at day 4, day 8, month 6, and month 12 (n=29), median values were broadly similar to the overall cohort (Table S6).

### Complete TMA Response

By 6 months after the index date, 47 (59.5%) patients had achieved complete TMA response. This increased to 54 (68.4%) within 12 months after the index date. The median (IQR) time to complete TMA response was 3.1 (1.0-14.0) months. Among the 3 patients who received a kidney transplant before initiating ravulizumab, complete TMA response was achieved in 2 patients; 1 patient by 6 months after the index date, and in 1 patient by 12 months after the index date.

### Dialysis Outcomes

Of the 57 patients who did not receive dialysis at baseline, 2 (3.5%) initiated dialysis during the time period from 2 weeks to 12 months after the index date (median [IQR] duration of 2.6 [1.1-4.1] months); both were off dialysis by the end of the observation period (month 12). Among the 20 patients who received any dialysis during the 12 months before the index date to 2 weeks after the index date, 14 (70.0%) did not have any dialysis reported during the period from 2 weeks to 12 months after the index date and 5 (25.0%) reported receiving dialysis during this period (median [IQR] duration of 0.6 [0.5-3.3] months). One (5.0%) patient had an unknown dialysis status during follow-up. Of the 11 patients requiring acute dialysis during the 12 months before the index date to 2 weeks after the index date, only 3 (27.3%) continued to receive acute dialysis during the 2 weeks to 12 months after the index date and all patients were off dialysis by the end of the observation period. For these 11 patients who received acute dialysis at baseline, the median (IQR) duration was 1.7 (0.4-2.4) months. Of the 8 patients requiring chronic dialysis during the 12 months before the index date to 2 weeks after the index date, 6 (75%) patients were off dialysis during follow-up, 1 patient received acute dialysis (and was off dialysis at the end of the observation period), and 1 patient received chronic dialysis during the period from 2 weeks to 12 months after the index date. For these 8 patients who received chronic dialysis at baseline, the median (IQR) duration was 3.6 (3.5-5.5) months.

## Discussion

This large study of 79 patients with aHUS aimed to address the need for real-world evidence of the effectiveness of ravulizumab among complement inhibitor-naive adults. Significant improvement in clinical endpoints, including hematologic and renal parameters, was observed from day 4 or 8 and was sustained to the last study timepoint (month 12).

The findings of this study may be considered in the context of a phase 3 trial of ravulizumab in a population of 56 complement inhibitor-naive adults with aHUS.^12^ Of note, the median time from first aHUS symptoms to the first dose of ravulizumab was 0.3 months in both studies. Both the current real-world study and the phase 3 trial demonstrated improvements in clinical endpoints after ravulizumab initiation, irrespective of differences in the study populations (eg, eligibility difference; patients with a variety of triggers could be included in the current study, yet they were excluded from the phase 3 trial).^12^ This study reported data from early timepoints after ravulizumab initiation (day 4) that suggest immediate improvement in clinical parameters. These data are further supported by case reports of ravulizumab treatment in patients with aHUS naive to C5 inhibitors^15^, ^16^, ^17^ and by analyses in smaller cohorts.^20^ Taken together, these data from C5 inhibitor-naive patients build a robust evidence base for making informed decisions about the use of ravulizumab in this patient population. Furthermore, real-world data indicate that patients with aHUS can successfully switch from eculizumab to ravulizumab treatment without any unexpected safety implications, and with maintained kidney function and hematologic parameters.^21^, ^22^, ^23^ Notably, a recent survey of adult patients and caregivers of pediatric patients with aHUS demonstrated a preference for ravulizumab over eculizumab, primarily driven by infusion frequency, which could be another consideration for treating physicians.^24^

The complete TMA response definitions were the same in the phase 3 trial and this study.^12^ In the phase 3 trial, 53.6% of patients met the primary endpoint of complete TMA response in 26 weeks, and the median time to complete TMA response was 86.0 days (∼2.8 months).^12^ The proportion of patients achieving complete TMA in 6 months was slightly higher (59.5%) in the current study with a similar median time (3.1 months). Of note, in the phase 3 trial, 60.7% of patients achieved complete TMA response at 2 years.^25^ There were some differences in demographic characteristics compared with the phase 3 study. For example, in the clinical trial, 52% of patients had received dialysis within 5 days of the first dose, whereas in the current study, only 25% had received dialysis within the 12 months before the first dose through to 2 weeks after. Furthermore, the clinical trial included 8 (14.3%) patients with a previous kidney transplant,^12^ to be compared with 3 (3.8%) patients in the current study.

Data from the Global aHUS Registry have indicated that a high proportion of patients with aHUS have a known trigger or an associated condition.^26^ Of note, in the current chart review study, 72% (n=47/65) of patients for whom trigger event data were available had an identifiable trigger event. This indicates that future studies may be warranted to investigate the clinical outcomes and the effectiveness of ravulizumab in subgroups of patients with triggering events to optimize the management of these patients in clinical practice.

A number of potential limitations should be considered when interpreting the data from this study. There are limitations inherent to the retrospective and observational design of the study, such as heterogeneity in data collection between different physicians/sites and missing data. For example, although the normal ranges used in this study aligned with those used in clinical trials of ravulizumab,^12^ they may differ from those used locally by some physicians/sites and thus not align with their designation for “normalization” of laboratory values. Data in patient charts were not originally collected for research purposes, such that detailed information may not be available for all the study variables. These issues were mitigated to a certain extent through the use of a standardized, pilot-tested electronic case report form with data validation checks. Furthermore, a sensitivity analysis focusing on patients without missing data for platelet counts, LDH levels, and SCr levels across day 4, day 8, month 6, and month 12 showed broadly similar trends to those of the overall study, indicating that any missing data had a limited effect. The inclusion criteria specified a requirement for 6 months of follow-up after the index date, which may have introduced a level of selection bias if a certain subset of patients were excluded more than others, such as those with minor or severe disease manifestations. Importantly, the study was double blinded and patient selection by physicians was randomized to reduce potential selection bias. To protect physician and patient anonymity, collected data could not be audited against medical records; therefore, queries on the accuracy of data entries could not be conducted. Of note, safety data were not collected during the study that could be assessed in parallel with the real-world efficacy data; however, ravulizumab has an established favorable safety profile from clinical trials with adults and pediatric patients.^12^, ^13^, ^14^^,^^25^ Furthermore, the only genetic information that was collected indicated whether testing had been performed, and if a mutation was detected in a given gene. The absence of details on specific variants limited the interpretation of the testing results, particularly with respect to pathogenicity. Finally, for patients with aHUS, triggering conditions are often necessary for aHUS manifestation and differential diagnoses may take time; therefore, some patients included in this study may have had TMAs unrelated to complement dysregulation^12^ but met the inclusion criteria at the time of the study. Because the data collected did not include detailed genetic variant information, it was not possible to discern insights into potential triggering events, such as autoimmune conditions. Future studies are warranted to further investigate genetic variants and triggers.

Physician panel-based studies such as this one also feature various scientific merits, including accessibility to a large panel of physicians from diverse geographic locations, broad representation of physicians in clinical practice, timeliness of real-world data, and a streamlined ethics review approval process.

In conclusion, this study provides real-world evidence to support the immediate and sustained benefits of initiating ravulizumab in patients with aHUS, as seen by the early response and continued improvement in clinical outcomes. Improvements in clinical endpoints, including hematologic and renal parameters, were observed from day 4 or 8 and were sustained to the last study timepoint (month 12).
