# Supplementary material for: Real-World Effectiveness of Ravulizumab Among C5 Inhibitor-Naive Patients With Atypical Hemolytic Uremic Syndrome: A Physician Panel-Based Chart Review (aHUS IMPACT Study)
Source: Kidney Med. 2025 Dec 9;8(2):101198. doi: 10.1016/j.xkme.2025.101198 (PMC12861229; doi:10.1016/j.xkme.2025.101198)
Supplement: Supplementary File (PDF) — Tables S1-S6. [file mmc1.pdf]

**Table S1. Physician characteristics**

| Characteristic | All physicians (N = 31) |
|---|---|
| **Medical specialty, n (%)** | |
| Hematologist-oncologist | 19 (61.3) |
| Nephrologist | 8 (25.8) |
| Hematologist | 4 (12.9) |
| **Years of practice, n (%)** | |
| 1–5 years | 1 (3.2) |
| 6–10 years | 4 (12.9) |
| 11–20 years | 18 (58.1) |
| ≥ 21 years | 8 (25.8) |
| **Clinical setting, n (%)** | |
| Office-based | 9 (29.0) |
| Hospital-based | 9 (29.0) |
| Mixed (office- and hospital-based) | 13 (41.9) |
| **Practice setting, n (%)** | |
| Academic | 17 (54.8) |
| Community | 14 (45.2) |
| **Practice size, n (%)** | |
| Solo (1 physician) | 2 (6.5) |
| Small (2–10 physicians) | 6 (19.4) |
| Medium (11–50 physicians) | 16 (51.6) |
| Large (≥ 51 physicians) | 7 (22.6) |
| **Geographic region,[a] n (%)** | |
| South | 10 (32.3) |
| West | 7 (22.6) |
| Midwest | 8 (25.8) |
| Northeast | 6 (19.4) |

Owing to rounding, some percentages do not sum to 100.
[a] The region categories followed those of US census. Midwest included IL, IN, IA, KS, MI, MN, MO, NE, ND, OH, SD, WI; Northeast included CT, ME, MA, NH, NJ, NY, PA, RI, VT; South included AL, AR, DE, DC, FL, GA, KY, LA, MD, MS, NC, OK, SC, TN, TX, VA, WV; West included AK, AZ, CA, CO, HI, ID, MT, NV, NM, OR, UT, WA, WY.
aHUS, atypical hemolytic uremic syndrome.

**Table S2. Summary of aHUS trigger events**

| **Event** | **All patients (N = 79)** |
|---|---|
| **Any trigger events, n (%)** | **47 (59.5)** |
| Autoimmune disease | 20 (42.6) |
| Infections from viruses or bacteria | 18 (38.3) |
| Malignant hypertension | 9 (19.1) |
| Drug | 6 (12.8) |
| Cancer | 4 (8.5) |
| Bone marrow transplant | 4 (8.5) |
| Solid organ transplant | 1 (2.1) |
| **None** | **18 (22.8)** |
| **Unknown** | **14 (17.7)** |

Categories are not mutually exclusive and may therefore sum to more than 100%.

**Table S3. Summary of genetic mutations among patients with an autoimmune trigger event**

| Patients with genetic mutations, n (%) | Patients with an autoimmune trigger event (N = 20) |
|---|---|
| **Patients for whom genetic tests were performed** | 18 (90.0) |
| **Any genetic variants detected[a]** | 15 (83.3) |
| Complement factor H (*CFH*) | 10 (66.7) |
| Complement factor H-related protein 1 (*CFHR1*) | 7 (46.7) |
| Complement factor H-related protein 5 (*CFHR5*) | 7 (46.7) |
| Membrane cofactor protein (*CD46*) | 5 (33.3) |
| Thrombomodulin (*THBD*) | 4 (26.7) |
| Complement factor B (*CFB*) | 4 (26.7) |
| Complement factor I (*CFI*) | 2 (13.3) |
| Complement factor H-related protein 3 (*CFHR3*) | 2 (13.3) |
| Complement component 3 (*C3*) | 2 (13.3) |
| **No genetic tests performed** | 2 (10.0) |

[a]Complement genetic variant testing (whole-exome sequencing) information was collected following aHUS diagnosis. Only the reporting of the following genetic variants was requested in the electronic case report form: *CD46*, *C3, THBD*, *CFHR1*, *CFHR3*, *CFHR5*, *CFH*, *CFI*, and *CFB*.

## Table S4. Summary of reasons for discontinuation of ravulizumab

| **Discontinued ravulizumab, n (%)[a]** | **20 (28.2)** |
|---|---|
| **Reasons for ravulizumab discontinuation, n (%)[b,c]** | |
| Reached optimal therapy length | 10 (50.0) |
| Additional reason(s) for treatment discontinuation | 9 (90.0) |
| Stabilization or normalization of renal function | 8 (80.0) |
| Physician decision | 4 (40.0) |
| Stabilization or normalization of renal function | 9 (45.0) |
| Additional reason(s) for treatment discontinuation | 8 (88.9) |
| Reached optimal therapy length | 8 (88.9) |
| Physician decision | 3 (33.3) |
| Physician decision | 7 (35.0) |
| Additional reason(s) for treatment discontinuation | 4 (57.1) |
| Reached optimal therapy length | 4 (57.1) |
| Stabilization or normalization of renal function | 3 (42.9) |
| Economic burden of treatment | 1 (5.0) |
| Disease progression | 1 (5.0) |
| Death | 0 (0.0) |
| Lack of access to treatment because of insurance/hospital policy | 0 (0.0) |
| Patient decision | 0 (0.0) |
| Pregnancy | 0 (0.0) |
| Other | 0 (0.0) |

[a]Proportion of patients with discontinuation data (N = 71).
[b]Proportion of patients who discontinued ravulizumab (n = 20).
[c]Reasons for ravulizumab discontinuation were recorded by physicians from a pre-defined list. The categories were not mutually exclusive and may add up to more than 100%.

**Table S5. Summary of laboratory values**

| Laboratory parameter (N = 79) | Baseline[a] | Time after index | | | | | | |
|---|---|---|---|---|---|---|---|---|
| | | 4 days | 8 days | 15 days | 1 month | 3 months | 6 months | 12 months |
| **Platelet count, × $10^9$/L** | | | | | | | | |
| n | 79 | 67 | 59 | 69 | 74 | 65 | 68 | 48 |
| Median | 87.0 | 110.0 | 120.0 | 147.0 | 160.0 | 200.0 | 210.0 | 229.0 |
| IQR | 37.0, 125.0 | 66.0, 145.0 | 97.0, 151.0 | 112.0, 171.0 | 132.0, 190.0 | 156.0, 223.0 | 168.0, 244.0 | 176.5, 285.0 |
| **LDH level, U/L** | | | | | | | | |
| n | 77 | 58 | 51 | 61 | 61 | 55 | 58 | 38 |
| Median | 370.0 | 315.0 | 301.0 | 277.0 | 235.0 | 222.0 | 187.5 | 122.0 |
| IQR | 310.0, 610.0 | 275.0, 450.0 | 222.0, 375.0 | 190.0, 334.0 | 134.0, 275.0 | 134.0, 261.0 | 112.0, 231.0 | 90.0, 169.0 |
| **SCr level, mg/dL** | | | | | | | | |
| n | 79 | 65 | 58 | 68 | 70 | 64 | 67 | 48 |
| Median | 2.2 | 2.2 | 1.9 | 1.6 | 1.5 | 1.3 | 1.1 | 1.0 |
| IQR | 1.7, 3.9 | 1.7, 3.7 | 1.6, 3.0 | 1.4, 2.2 | 1.2, 1.8 | 1.0, 1.7 | 0.9, 1.3 | 0.9, 1.4 |
| **eGFR, mL/min/1.73 $m^2$** | | | | | | | | |
| n | 75 | 48 | 45 | 52 | 53 | 50 | 49 | 28 |
| Median | 36.0 | 45.0 | 51.0 | 60.5 | 65.0 | 76.0 | 81.0 | 70.0 |
| IQR | 20.0, 49.0 | 28.0, 55.0 | 35.0, 65.0 | 45.0, 69.5 | 50.0, 78.0 | 56.0, 87.0 | 60.0, 92.0 | 64.5, 79.0 |
| **Hemoglobin level, g/L** | | | | | | | | |
| n | 78 | 66 | 58 | 67 | 70 | 64 | 68 | 47 |
| Median | 85.0 | 90.5 | 100.0 | 107.0 | 110.0 | 120.0 | 120.5 | 120.0 |
| IQR | 80.0, 105.0 | 83.0, 110.0 | 90.0, 111.0 | 93.0, 117.0 | 100.0, 120.0 | 101.5, 130.0 | 110.0, 130.0 | 105.0, 130.0 |

[a]Baseline laboratory values were collected on index date or at a point closest to the index date within the previous 6 months.
eGFR, estimated glomerular filtration rate; IQR, interquartile range; LDH, lactate dehydrogenase; SCr, serum creatinine.

**Table S6. Summary of laboratory values in patients without missing data for platelet count and LDH and SCr levels across Day 4, Day 8, Month 6, and Month 12**

| Laboratory parameter (n = 29) | Baseline[a] | Time after index | | | | | | |
|---|---|---|---|---|---|---|---|---|
| | | 4 days | 8 days | 15 days | 1 month | 3 months | 6 months | 12 months |
| **Platelet count, × $10^9$/L** | | | | | | | | |
| n | 29 | 29 | 29 | 29 | 29 | 29 | 29 | 29 |
| Median | 87.0 | 99.0 | 125.0 | 150.0 | 177.0 | 211.0 | 229.0 | 265.0 |
| IQR | 45.0, 122.0 | 75.0, 140.0 | 100.0, 152.0 | 123.0, 182.0 | 150.0, 196.0 | 178.0, 245.0 | 190.0, 312.0 | 210.0, 321.0 |
| **LDH level, U/L** | | | | | | | | |
| n | 29 | 29 | 29 | 29 | 29 | 29 | 29 | 29 |
| Median | 555.0 | 389.0 | 263.0 | 222.0 | 170.0 | 145.0 | 155.0 | 131.0 |
| IQR | 334.0, 678.0 | 290.0, 460.0 | 199.0, 356.0 | 167.0, 333.0 | 123.0, 245.0 | 98.0, 230.0 | 90.0, 211.0 | 90.0, 190.0 |
| **SCr level, mg/dL** | | | | | | | | |
| n | 29 | 29 | 29 | 29 | 29 | 29 | 29 | 29 |
| Median | 3.5 | 3.0 | 2.0 | 1.7 | 1.5 | 1.1 | 1.0 | 1.0 |
| IQR | 2.0, 5.6 | 1.9, 4.0 | 1.6, 3.0 | 1.0, 2.3 | 1.0, 1.8 | 1.0, 1.5 | 0.9, 1.3 | 0.8, 1.2 |
| **eGFR, mL/min/1.73 $m^2$** | | | | | | | | |
| n | 28 | 23 | 23 | 23 | 23 | 23 | 23 | 23 |
| Median | 31.5 | 33.0 | 40.0 | 50.0 | 55.0 | 56.0 | 68.0 | 70.0 |
| IQR | 13.5, 47.5 | 17.0, 56.0 | 33.0, 68.0 | 38.0, 68.0 | 49.0, 72.0 | 51.0, 76.0 | 56.0, 85.0 | 64.0, 81.0 |
| **Hemoglobin level, g/L** | | | | | | | | |
| n | 28 | 29 | 28 | 29 | 28 | 29 | 29 | 29 |
| Median | 80.0 | 90.0 | 99.5 | 100.0 | 110.0 | 120.0 | 130.0 | 129.0 |
| IQR | 78.0, 90.0 | 80.0, 100.0 | 90.0, 100.0 | 94.0, 110.0 | 100.0, 120.0 | 102.0, 130.0 | 106.0, 134.0 | 113.0, 136.0 |

[a]Baseline laboratory values were collected on index date or at a point closest to the index date within the previous 6 months.
eGFR, estimated glomerular filtration rate; IQR, interquartile range; LDH, lactate dehydrogenase; SCr, serum creatinine.
